# Improving the Quality of Operative Notes in Vascular Surgery: A Retrospective Analysis

**DOI:** 10.7759/cureus.54791

**Published:** 2024-02-23

**Authors:** Munzir Akasha, Ahmmad Alfatih, Mohamedali Mohamed, Yogesh Acharya, Mahmoud Alawy

**Affiliations:** 1 Department of Vascular Surgery, University Hospital Galway, Galway, IRL; 2 Department of Cardiothoracic Surgery, University Hospital Galway, Galway, IRL

**Keywords:** quality measure, evaluation, healthcare, quality improvement, operative note

## Abstract

Background and objective

Missing information or mistakes in patients’ medical records, including those related to intraoperative and postoperative information, in an operative note can have profound clinical, ethical, and medicolegal implications. Operative notes should be informative, clear, and inclusive of the necessary data and should be collated immediately following surgery. In this study, we aimed to determine the ways to improve the quality of operative notes in the field of vascular surgery.

Methods

In this retrospective analysis, we compared the operative notes of 32 patients in the Department of Vascular and Endovascular Surgery, University Hospital Galway, against the standard set by the Royal College of Surgeons in Ireland (RCSI) (Code of Practice for Surgeons RCSI, 2018) and presented the results to our departmental staff. To facilitate an improvement in the quality of operative notes, a structured poster checklist was designed and displayed in the operating theatre. Furthermore, a scanner was set up in the operating theatre with clear and easy-to-follow instructions for uploading the operative notes into our hospital’s online and digital patient record system (EVOLVE). An explanatory video was circulated among the staff. Three months after the first cycle, two further retrospective cycles were performed.

Results

A total of 96 patients’ operative notes were analysed. Following the intervention, a significant improvement in documentation was noted concerning the dates; procedures followed; as well as the details of surgeons, assistants, anesthetists, incisions, surgery types, operative diagnoses, complications, additional procedures, tissue details, prostheses involved, closure techniques, postoperative plans, and surgeons’ signatures. We also observed a significant increase in the uploading of the operative notes in the EVOLVE system.

Conclusions

The quality of the operative notes improved considerably after staff education, poster display, and scanner installment in the operating theatre. It is important to have an efficient and well-structured plan to improve the process of operative note-keeping, thereby ultimately enhancing overall patient care.

## Introduction

The Department of Vascular Surgery at the University Hospital Galway offers round-the-clock, seven-day-a-week tertiary service for vascular patients. The on-board staff consists of four consultants, five registrars, and four senior house officers catering to patients in the western region of Ireland. The standard operative notes at University Hospital Galway are usually handwritten by the consultant or registrar using their preferred method of documentation. This has resulted in many discrepancies, either due to the failure to record relevant information or unnecessary documentation. During postoperative outpatient follow-up, most patients are provided with new temporary charts. The lack of operative notes on the EVOLVE system results in delays in providing medical care at the outpatient department of the hospital.

Surgeons’ practices are guided by a code of practice and ethical obligations. The Code of Practice for Surgeons at the Royal College of Surgeons in Ireland (RCSI) document outlines the necessary steps to be taken for record-keeping, which includes clear, readable operative notes containing the details of comprehensive postoperative patient care that should be readily available throughout the patient’s recovery course [1]. Missing information or mistakes in patients’ medical records, including those related to intraoperative and postoperative documentation, in an operative note can lead to potential clinical, ethical, and medicolegal issues [2]. Hence, operative notes should be informative, clear, and inclusive of all necessary data and should be collated immediately after surgery [3,4]. Adequate documentation of operative notes is vital since the notes are often used for research, audits, risk management, and educational purposes [5]. Although digital documentation provides more accurate operative details than handwritten documentation [6], its implementation is difficult. Against this backdrop, this study aimed to compare vascular operative notes at University Hospital Galway against RCSI standards.

## Materials and methods

The QI team comprised a consultant, registrar, and senior house officers. Initially, we performed a retrospective analysis of 32 patients’ operative notes with vascular conditions in October 2021 (first cycle). The data were extracted in an Excel spreadsheet and compared against RCSI standards, including information on dates and times of the procedure, whether the procedures were elective or emergency procedures, names of the surgeons and assistants, anesthetists, the procedures, incisions, operative diagnoses, operative findings, complications, additional procedures, tissue alterations or removals, prostheses used, closure techniques, anticipated blood loss, antibiotic prophylaxes, postoperative care instructions, and surgeons’ signature. Additionally, we checked the availability of the operative notes on the EVOLVE system. Following an initial baseline measurement, we completed two additional cycles, with three-month intervals between each cycle. A total of 96 patients' operative notes were included in the study.

**Figure 1:**
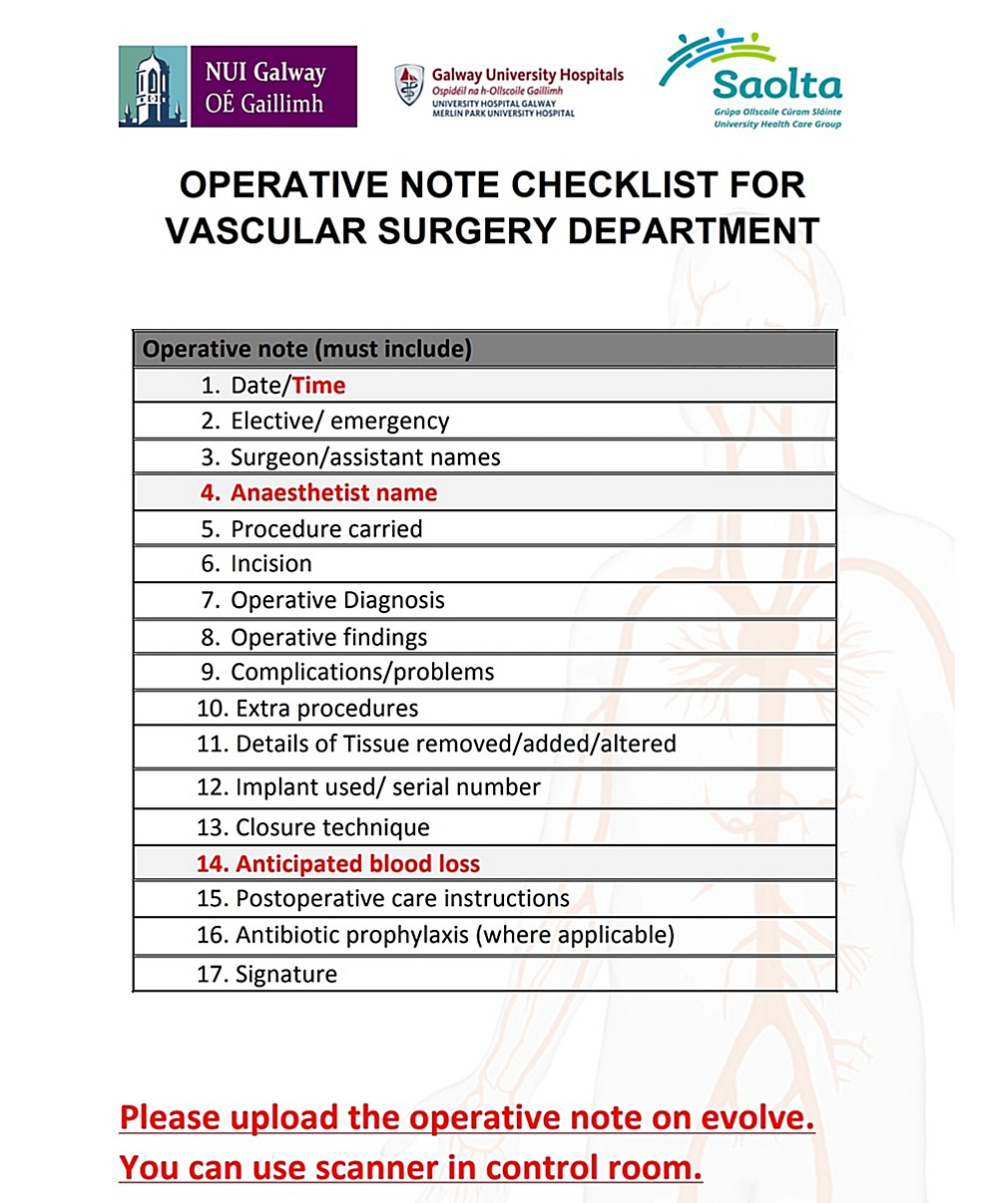
Theatre checklist poster

Following careful discussion, we decided to display a poster as a reminder and install a scanner in the operating theatre. A poster was designed to quickly recall all the documentation aspects to enable documentation of all the required information along with the frequently overlooked details while recording the baseline measurements in the operative notes (Figure 1). In addition, a scanner was installed next to the operating theatre as a simple method to upload operative notes to the system. Because we anticipated that some staff members would face technical difficulties while using the scanner, we created a simple explanatory video that was shared across the department.

We presented the findings of our baseline measurements at the departmental morbidity and mortality meetings. We simultaneously educated the staff about the importance of comprehensive documentation of operative notes. They were also informed about the installation of the scanner and the steps required for uploading the operative notes. This resulted in an overall improvement in the uploading of the operative notes. However, some staff still reported difficulty using the scanner, especially since individual account setting was required.

**Figure 2:**
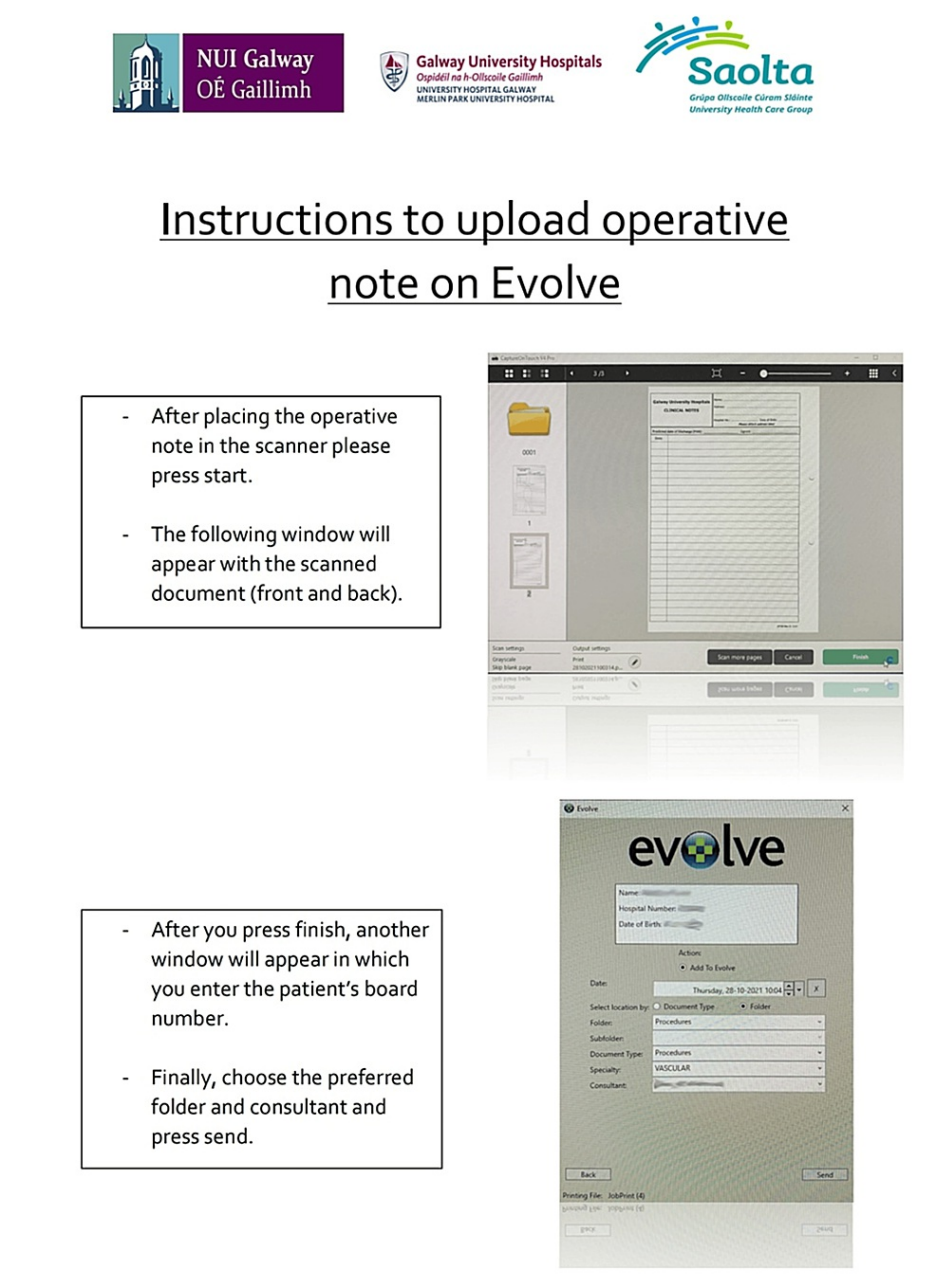
EVOLVE upload instruction poster

A team of two senior house officers was involved in setting the accounts of the departmental staff to access the scanner. Additionally, an explanatory poster was designed and placed next to the scanner (Figure 2). The results of the second cycle were presented, and staff members were re-educated about the significance of record keeping.

## Results

Overall, a total of 96 patients’ operation notes were reviewed over six months. All the notes were handwritten. The baseline assessment revealed an acceptable level of documentation for the procedure names (32/32, 100%), postoperative instructions (32/32, 100%), names of the surgeons and assistants (32/32, 100%), surgeons’ signatures (32/32, 100%), operative diagnoses (31/32, 96.9%), operative findings (31/32, 96.9%), and incisions (28/32, 87%). Twenty-four implants were performed, and implant identification was noted in 91.6% (22/24) of cases. In contrast, poor outcomes were noted for antibiotic prophylaxes and anticipated blood loss (0%, 0/32), complications (8/32, 25%), the urgency of procedure (3/32, 9.4%), additional procedures performed (4/32, 12.5%), anesthetist name (19/32, 59%), details of tissue removed or altered (13/32, 40%), closure techniques (19/32, 59%), and availability of the operative note on the EVOLVE (9/32, 28.1%) system (Table 1). Furthermore, the dates were recorded for all operative notes (32/32, 100%); however, documentation of the time of the procedure was completely neglected.

**Table 1:** Results of the data documented over three cycles

Standard parameters	1^st^ cycle	2^nd^ cycle	3^rd^ cycle
Date	100%	100%	100%
Time	0%	28.1%	6.25%
Elective/emergency	9.4%	46.8%	50%
Name of procedure	100%	100%	100%
Name of surgeon (S) and assistants	100%	100%	100%
Name of anesthetist (S)	59%	71.8%	90.6%
Incision	87%	84.3%	100%
Operative diagnosis	96.8%	96.8%	100%
Operative findings	96.9%	100%	100%
Problems or complications	25%	40.6%	90.6%
Additional procedures	12.5%	43.7%	75%
Details of tissue implanted/removed/altered	40.6%	37.5%	71.8%
ID for prosthesis and other implant materials present	68.8%	93.7%	100%
Closure technique	59.4%	81.2%	100%
Anticipated blood loss	0%	34.3%	9.3%
Prophylactic antibiotics	0%	43.7%	28.1
Postoperative plan	100%	100%	100%
Surgeons’ signature	100%	100%	100%
Uploaded on EVOLVE	28.1%	62.5%	90.6%

In the final cycle, the documentation of the following data improved with a frequency of 100%: date, name of the procedure, name of surgeon and assistant, operative diagnosis and findings, incision, prosthesis identification, closure technique, postoperative plan, and surgeons’ signature. In addition, the documentation of other data also improved, including the type of surgery (50%), name of anesthetist (90.6%), complications (90.6%), additional procedures (75%), tissue details (71.8%), and uploading the operative note on the EVOLVE system (90.6%). Although documentation of time, anticipated blood loss, and antibiotic prophylaxis increased from 0% to 28.1%, 34.3%, and 43.7%, respectively, in the second cycle, they were documented less frequently in the third cycle.

## Discussion

Operative notes are crucial documents in the medical field, providing a detailed account of surgical procedures. They serve as a comprehensive surgery record that can aid in postoperative care, research, and legal matters. The accuracy and completeness of these documents depend on the quality of operative note writing.

In our study, the documentation quality markedly improved in most aspects and has been maintained within an acceptable range (Table 1) after installing the scanner in the operation theatre and displaying the poster. The strongest aspect of our study was the strategic plan implemented based on the feedback from concerned stakeholders. Despite some people reporting technical difficulties, even after providing an explanatory video for operating the scanner, we conducted personal training and scanner account registration, which increased the number of operative notes that were uploaded. Throughout the three observation cycles, we noticed a significant improvement in most documentation processes. However, one issue that we need to focus on is the amount of anticipated blood loss, especially in a vascular theatre. This factor can have a significant impact on the postoperative course, and it can vary depending on the type of procedure, setting (elective or emergency), and the patient's preoperative condition in terms of anticoagulation.

We reviewed the existing literature for further comparison with our findings. A study by Khalid et al. (September 19, 2022) [7] involved a similar issue at a tertiary hospital in Lahore and documented the following results after completing the audit (against the results from our study). Date of procedure: 100% vs. 100% in our study; time of the procedure: 87.5% vs. 6.25%; elective/emergency procedure: 100% vs. 50%; the name of surgeon and assistant: 100% vs. 100%; the name of the operative procedure: 97.9% vs. 100%; type of incision: 95.8% vs. 100%; operative diagnosis: 97.9% vs. 100%; operative findings: 95.8% vs. 100%; complications encountered: 93.8% vs. 90%; any extra procedure performed with reason: 93.8% vs. 75%; details of tissue removed, added, or altered: 93.8% vs. 71.8%; details of closure technique: 95.8% vs. 100%; anticipated blood loss: 95.5% vs. 9.3%; antibiotic prophylaxis: 97.9% vs. 28.1%; DVT prophylaxis: 60.4% vs. NA; detailed postoperative care instructions: 91.7% vs. 100%; and signature: 100% vs. 100%.

Another study conducted by Lim and Wong in 2022 [8] evaluated the quality of operative notes in a vascular surgery department. According to their report, the compliance rate for recording the date and the name of the operator was 97.5%, while that for the name of the anesthetist, operative procedure, and closure technique was 100%. However, they observed that the following areas required improvement: time (0%), anticipated blood loss (2.5%), and elective/emergency procedures (10%) in the first cycle; by the end of the second audit cycle, a standardised operative note sheet was used, resulting in a 100% compliance rate in all aspects of the audit.

Theivendran et al. [9] implemented an electronic record system, which significantly improved the efficiency and quality of operative notes. Before the implementation of the electronic template, the average time taken to type the notes was 11.6 days (range: 7-22 days), and adherence to RCS guidelines was 71.1% (range: 63-72%). However, after the introduction of the electronic template, the notes were typed immediately after the procedure and printed out into the patient's notes, resulting in zero delays. The adherence to RCS guidelines also improved significantly to 91%. Further refinements, such as including the DVT prophylaxis documentation, led to 100% compliance with no delays in typing the operative notes. Overall, the electronic record system helped to save time and improve the quality of the operative notes.

Limitations

This study has a few limitations. While the hospital utilises an electronic system for the majority of the documentation, in certain entities, the classical handwritten notes are still in use, and the operative notes constitute one of those entities; the sheet has preprinted subheadings including patient addressograph label, operation name, operating surgeon, anesthetist, and scrub nurse names. The rest of the sheet is blank and the information has to be manually entered. It was difficult in some cases to read the handwriting of some of our colleagues. Providing an electronic record with a dropdown list can help tackle this issue.

## Conclusions

Our intervention led to a significant increase in the number of operative notes uploaded, which rose to 90.6%. We attribute this improvement to the provision of clear instructions to the surgical staff. However, we acknowledge that financial constraints and limited resources could still be a hindrance to the implementation of electronic records, which are more efficient than handwritten notes. To overcome these challenges, we recommend that all surgical specialties adopt the standard code of practice. Additionally, regular training sessions should be provided to the staff to ensure that all operative notes are available in digital format. This will not only improve the efficiency of the documentation process but also reduce the likelihood of errors.
